# Complete mitochondrial genome sequencing and identification of candidate genes responsible for C5-type cytoplasmic male sterility in cabbage (*B. oleracea* var. *capitata*)

**DOI:** 10.3389/fpls.2022.1019513

**Published:** 2022-09-26

**Authors:** Xionghui Zhong, Xiangqing Yue, Jian Cui, Rui Han, Yi Gao, Jungen Kang

**Affiliations:** ^1^ Beijing Vegetable Research Center, Beijing Academy of Agriculture and Forestry Sciences, Key Laboratory of Biology and Genetic Improvement of Horticultural Crops (North China), Ministry of Agriculture, Beijing, China; ^2^ College of Horticulture, Gansu Agricultural University, Lanzhou, China

**Keywords:** cytoplasmic male sterility (CMS), ORF222a, ATP17, mitochondrial genome, tapetal cell

## Abstract

Cytoplasmic male sterility (CMS) is widely used in cruciferous vegetables hybrid breeding. The C5-type CMS cabbage line exhibits stable male sterility and offers great value for cabbage breeding. However, the underlying CMS mechanism remains unclear. Here, the complete mitochondrial genome was sequenced and assembled for this line. The genome size was 221,862 bp. Mitochondrial genome comparison showed that the mitochondrial genome was likely generated by recombination with a *nap*-type CMS *B. napus* strain. Sixty-seven unknown-function open reading frames (ORFs) were identified. Seven *orfs*, *orf114a*, *orf123a*, *orf188a*, *orf*222a, *orf*261a, *orf286a*, and *orf322a*, were specifically identified in this genome. The presence of these candidate CMS genes decreased ATPase activity and ATP content by affecting the transcript levels of energy metabolism-related genes and F_1_F_0_-ATP synthase assembly. Among them, *orf188a*, *orf222a*, *orf261a*, *orf286a*, and *orf322a* possessed a transmembrane structure, and *orf188a* was cotranscribed with *rps7* and *trnfM*. *orf222a* was partially homologous to *atp8* and coexpressed with *nad5*. *orf261a* and *orf322a* were cotranscribed with *cox1* and *atp9*, respectively. Additionally, *orf114a* was cotranscribed with *atp8*. Yeast two-hybrid assays showed that the ORF222a protein interacts with a *B. oleracea* ATP17 homolog (*Bo7g114140*) during F_0_-type ATP synthase assembly, reducing the quantity and activity of assembled F_1_F_0_-ATP synthase. Cytological sections showed that premature separation of the tapetum from the connective tissue and delayed tapetal programmed cell death (PCD) might be the immediate causes of CMS in C5-type CMS cabbage lines. Our results may help uncover the molecular mechanism of C5-type CMS in *B. oleracea* from the perspectives of the whole mitochondrial genome and cytology of anther development.

## Introduction

Cabbage (*Brassica oleracea* var. *capitata*) is one of the most important vegetables and is grown on five continents around the world  (Chiang et al., 1993). In the last few years, approximately 70 million tonnes of cabbage have been produced per year worldwide; according to the Food and Agriculture Organization of the United Nations (FAO), China is the largest cabbage producer in the world, producing approximately 34 million tonnes in 2020. Heterosis has been widely applied in cereal crop and vegetable production (Yu et al., 2021). Cytoplasmic male sterility (CMS) is an extranuclear maternally transmitted trait that produces either aborted or infertile pollen grains (Dong et al., 2013). CMS lines, along with maintainer and restorer lines, are highly valuable resources for F1 hybrid seed production, which has been widely developed and applied to all *Brassicaceae* crops (Yamagishi and Bhat, 2014; Yang et al., 2021).

In *Brassica* species, a variety of CMS types have been independently reported, such as *Ogu*, *nap*, *Nig*, *tour*, *Shaan2A*, *pol*, *Nsa*, *hau*, *inap*, and *oxa*. Among them, Ogu-CMS was first identified in *Raphanus sativus* (Ogura, 1967); *Nig* CMS comes from *Brassica nigra* (Pearson, 1972); *nap* CMS, *Shaan2A* CMS and *pol* CMS are derived from *Brassica napus* (Shiga and Baba, 1971; Li, 1980; Fu, 1981); *Nsa* CMS is an alloplasmic male sterility system derived from somatic hybridization between *Brassica napus* and *Sinapis arvensis* (Hu et al., 2002); *inap* CMS was obtained from somatic hybridization between *Brassica napus* and* Isatis indigotica* by recurrent backcrossing (Kang et al., 2017); and *tour* CMS, *hau* CMS and *oxa* CMS originated from *Brassica juncea* (Rawat and Anand, 1979; Wan et al., 2008; Heng et al., 2018). The *Nig*, *tour*, *pol* and *Ogu* CMSs were transferred into *B. oleracea* by protoplast fusion or the interspecific cross; however, the cybrids exhibited poor agronomic performance except for Ogu-CMS system (Pearson, 1972; Bannerot H et al., 1974; Yarrow et al., 1990; Cardi and Earle, 1997). To date, the Ogu-CMS system has been used as the major CMS system for F1 seed production in *B. oleracea* crops because of its stable sterility and lack of adverse effects on plant growth (Dey et al., 2013). However, the broad use of a single CMS results in a high degree of cytoplasmic genetic uniformity, which may lead to genetically vulnerable plants (Yamagishi and Terachi, 2017). Therefore, it is urgent to develop more CMS systems for cabbage breeding.

Thus far, many CMS-related genes have been identified and reported in *Brassica* species. Most open reading frame (ORF)-encoded proteins in CMS lines disrupt energy metabolism, which disrupts the F_1_F_0_-ATP synthase subunit. The molecular structure of the F_1_F_0_-ATP synthase complex from *Saccharomyces cerevisiae* has already been built (Srivastava and Luo, 2018). However, the structure of this complex in plants has not been resolved. Plant F_1_F_0_-ATP synthase shares the basic structure of the enzyme complexes described in yeast. The water soluble complex (F_1_) component possesses 3 copies each of subunits α (ATP1) and β (ATP2) and one copy each of subunits γ (ATP3), δ (ATP16) and ϵ (ATP15). The membrane complex (F_0_) is composed of subunits a (ATP6-1 and ATP6-2), b (ATP4), 8 (ATP8), c (ATP9), f (ATP17), g (ATP20) and e (ATP21) (Zancani et al., 2020). Subunits a (ATP6), 8 (ATP8), and c (ATP9) have been extensively investigated in most CMS types. Several previous findings showed that products of candidate CMS-related genes often affect mitochondrial functions *via* interacting with the other essential nuclear/mitochondria-encoded proteins. For examples, ORFH79 impairs mitochondrial function *via* interaction with P61, which is a subunit of electron transport chain complex III in Honglian CMS rice line (Wang et al., 2013). The mitochondrial protein WA352 interacts with COX11 to inhibit its function in peroxide metabolism, which induces the premature tapetal PCD and triggers consequent pollen abortion in the Wild Abortive CMS (CMS-WA) rice line (Luo et al., 2013). ORF224 was found to be associated with a respiratory electron transport chain protein (BnaC03g14740D), which affects the development of anthers and induces pollen abortion in the *pol* CMS line of *B. napus* (Wang et al., 2021). Additionally, the CMS-related genes can also affect mitochondrial functions though cotranscribing with the essential mitochondrial genes. The sterility gene *orf138*, which triggers Ogu-CMS, is cotranscribed with *atp8* and *trnfM* (Tanaka et al., 2012; Zhong et al., 2021). The sterility gene *orf224* is responsible for *pol* CMS, which is cotranscribed with *atp6* (Singh and Brown, 1993). *orf222*, the key gene of *nap* CMS, is similar to the *pol* CMS-associated gene *orf224* and the cotranscribed genes *nad5c* and *orf139* (Lhomme et al., 1997). *orf288*, the master gene of *hau* CMS, is also cotranscribed with the downstream gene *atp6* (Heng et al., 2014). The key sterility gene *orf346*, which controls *Nsa* CMS, is cotranscribed with the *nad3* and *rps12* complex (Sang et al., 2021). The sterility gene *orf193*, which triggers *tour* CMS (originating from cell fusion between *B. napus* and *B. tournefortii*), is cotranscribed with the downstream gene *atp9-2* (Dieterich et al., 2003). However, *orf263*, the causal gene for *tour* CMS (obtained from sexual hybridization between *B. napus* and *B. tournefortii*), is cotranscribed with the downstream gene *atp6* (Landgren et al., 1996). In total, the expression of specific mitochondrial genes is highly dependent on the species in which the CMS source originated.

In the present study, C5-type CMS was identified as a novel alloplasmic male-sterility system derived from intergeneric hybridization of *B. napus* and *B. oleracea*. To provide new insights into the molecular mechanism of this C5-type CMS of cabbage exhibiting anther indehiscence and no pollen dispersal, we sought to identify CMS-associated candidate genes in mitochondria using a next-generation sequencing approach and accordingly analyzed the specific ORFs. The complete mitochondrial genomes of C5-type CMS and its maintainer line were sequenced. To deeply explore the abortive mechanisms of the C5-type CMS in cabbage, we investigated the characteristics of maintainer and C5-type CMS cabbage lines in cytological, physiological, and molecular analyses. To ascertain the responsible gene and the underlying mechanisms for C5-type CMS, we characterized C5-type CMS-associated candidate genes. The mitochondrial genome comparison results indicated that the C5-type CMS original cytoplasm donor material was most likely generated by recombination with the *nap-*type CMS *B. napus* strain through intergeneric hybridization during the breeding process. The CMS candidate protein ORF222a interacts with the ATP17 homolog (*Bo7g114140*) in *B. oleracea*, impairing the step of F_1_Fo-ATP synthase assembly, which results in remarkable reductions in the ATP and ATPase activity levels in the anther samples of C5-type CMS cabbage lines compared with those of the maintainer lines. Cytological analysis of anther development between the maintainer and C5-type CMS cabbage lines showed that premature separation of the tapetum from the connective tissue and abnormal degradation of tapetal cells might be the immediate cause of CMS in C5-type CMS cabbage lines.

## Materials and methods

### Plant materials

The maintainer line was an open-pollinating and early-maturing cabbage variety. The original cytoplasmic donor cabbage material was introduced from *B. napus nap*-type cytoplasm by intergeneric hybridization. The C5-type CMS cabbage line was obtained from crosses and consecutive backcrosses with *B. napus* in 2005 by Dr. Jungen Kang from the Beijing Vegetable Research Center of the Beijing Academy of Agriculture and Forestry Sciences (BAAFS). The stability of C5-type CMS was observed for more than 10 years. The mitochondrial DNA of both the C5-type CMS and maintainer lines was sent to Biozeron Company (Shanghai, China) for DNA library construction and sequencing in 2018.

### Mitochondrial genome sequencing and assembly

One microgram of purified mitochondrial DNA was fragmented for 300-500 bp paired-end library construction using a TruSeq™ Nano DNA Sample Prep Kit. Sequencing was performed on the Illumina HiSeq 4000 platform (BIOZERON Co., Ltd., Shanghai, China). A DNA library with approximately 15-20 kb SMRTbell libraries was constructed and sequenced on a PacBio Sequel Sequencer (PacBio Inc., Menlo Park, CA, USA). For the cabbage mitochondrial genome assembly, the filtered Illumina HiSeq subreads were preliminarily assembled by ABySS v2.0.2 software (version 1.5) (Jackman et al., 2017). Then, the PacBio Sequel data were aligned by the blasR method for single-molecule sequencing data correction. The corrected PacBio Sequel data and Illumina HiSeq data were combined for the mitochondrial genome framework assembly using SPAdes v3.10.1 software (Antipov et al., 2016). Finally, clean Illumina HiSeq reads were mapped to the assembled mitochondrial genome to verify the accuracy of the sequence. The circular genome maps were drawn using OrganellarGenomeDRAW (version 1.2) (Greiner et al., 2019).

### Gene prediction and annotation

Mitochondrial genes were predicted based on a combination of homology-based gene prediction and *de novo* prediction by Genewise and AUGUSTUS software. The tRNA and rRNA genes were predicted using tRNAscan-SE (version 2.0) and rRNAmmer (version 1.2), respectively (Lowe and Eddy, 1997; Lagesen et al., 2007). The functions of the predicted proteins were annotated based on a BLASTP search against universal databases, such as the National Center for Biotechnology Information (NCBI) database, the Gene Ontology (GO) database (Ashburner et al., 2000), the Evolutionary Genealogy of Genes: Non-supervised Orthologous Groups (eggNOG) database (Jensen et al., 2008), and the Kyoto Encyclopedia of Genes and Genomes (KEGG) database (Kanehisa et al., 2004). The transmembrane domains in each candidate ORF-encoded protein were assessed using TMHMM Server v.2.0 (http://www.cbs.dtu.dk/services/TMHMM/).

### ATP content and synthase activity measurement

The ATP content and synthase activity were measured in the anthers of the maintainer and C5-type CMS lines with an ATP content assay kit and a Na^+^K^+^-ATP synthase activity assay kit, respectively (Solarbio Co., Ltd., Beijing, China). The procedures were performed according to the manufacturer’s instructions (Solarbio, BC0300 and BC0065).

### Y2H assay

The full-length coding sequences (CDSs) of *atp4*, *atp6*, *atp8*, *atp9*, and two *atp17*-homologous genes (*Bo3g175820* and *Bo7g114140*) were cloned into pGADT7 vectors; *orf188a*, *orf222a* and *orf261a* were cloned into the PGBKT7 vector. The eighteen candidate interaction combinations were transformed into the yeast strain Y2HGold. Y2H assays were performed according to the Clontech manual. The transformed yeast cells were inoculated onto synthetic defined medium without leucine and tryptophan (SD/-Leu/-Trp) and incubated at 30°C for 2-3 days. The single transformation colony was then gradient-diluted and inoculated onto SD/-Leu/-Trp/-His-Ade media and cultured for 3–5 days at 30°C to observe strain growth.

### Cytological analysis

Flower buds from the maintainer and C5-type CMS lines were collected at six different developmental stages and immediately fixed in formalin-aceto-alcohol (FAA) solution. Paraffin sections were then prepared according to the method of Zhong et al. (2021). Here, anther transverse sections were stained in 1% toluidine blue solution and viewed under a Leica DMR2 microscope (Leica, Wetzlar, Germany), and images were obtained with a Nikon Coolpix4200 camera (Nikon, Tokyo, Japan)

### Cloning of CMS-associated candidate ORFs and subunit genes of F_1_Fo-ATP synthase

Total RNA was isolated from the anthers of maintainer and C5-type CMS lines using an RNA extraction kit (RNAprep Pure Plant Kit, Tiangen, Beijing, China). First-strand cDNA was synthesized using a PrimeScript™ RT Reagent Kit (Takara, RR037A, Dalian, China). Subunits b (*atp4*), a (*atp6*), 8 (*atp8*), and c (*atp9*) were cloned from cDNA of the C5-type CMS line; however, the subunit f (ATP17) homologs (*Bo3g175820* and *Bo7g114140*) were cloned from cDNA of the maintainer line. Cloning PCR was performed using the primers listed in **Supplementary Table S1**. The amplified PCR products were detected using gel electrophoresis (1.5% agarose gel).

### Alignment and phylogenetic analysis

Geneious Prime software was used to perform multiple sequence alignment between *B. napus* strain 51218 and our C5-type CMS mitochondrial sequences to obtain consistent sequences. Seven mitochondrial genome sequences of *B. napus* lines (*B. napus* strain SW18: AP018473 and AP018474, strain 51218: KP161618, cultivar 088018: MW348924, cultivar NY18: MW001149, and cultivar Westar: AP006444) were obtained from the NCBI database (https://www.ncbi.nlm.nih.gov/genome/browse/). Mitochondrial genome-wide alignments and ML bootstrap analysis with 500 replicates were performed using MEGA 6.0 (Tamura et al., 2013).

## Results

### Flower morphology of the C5-type CMS line and maintainer lines

**Figure 1:**
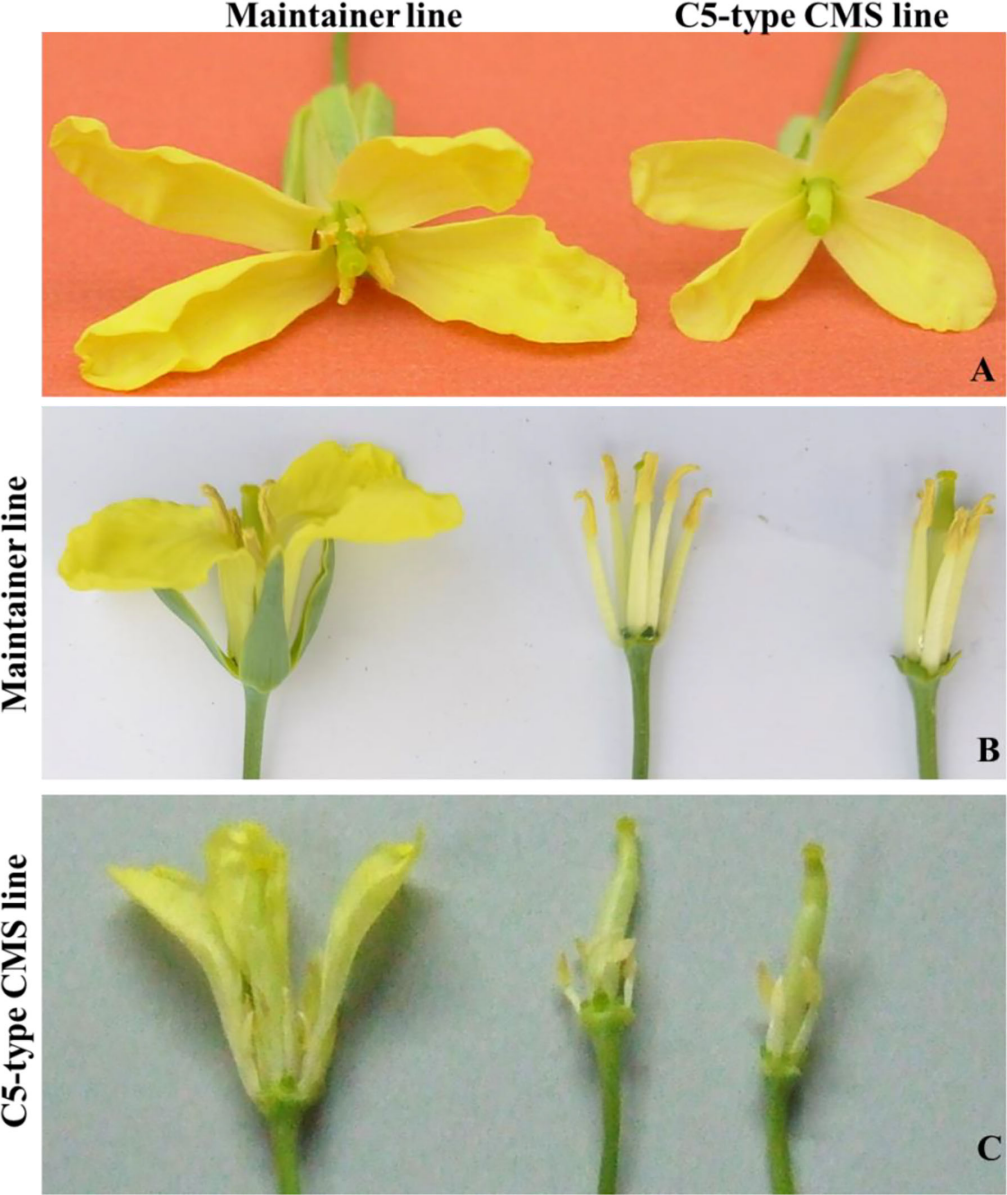
Flower morphology of maintainer lines and C5-type CMS lines. **(A)** Intact flowers of the C5-type CMS line and maintainer lines. **(B)** Partially dissected maintainer flower. **(C)** Partially dissected C5-type CMS flower.

The sterile flowers of the C5-type CMS line were significantly smaller than the fertile flowers of the maintainer line. The filaments and anthers of fertile flowers were remarkably longer than those of sterile flowers. There were no differences in the pistils between the C5-type CMS and maintainer lines. The C5-type CMS line exhibited a sterile phenotype due to anther indehiscence and no pollen dispersal. However, the maintainer line produced normal pollen grains (**Figure 1**).

### Mitochondrial genome sequencing and annotation of the C5-type CMS cabbage line

**Figure 2:**
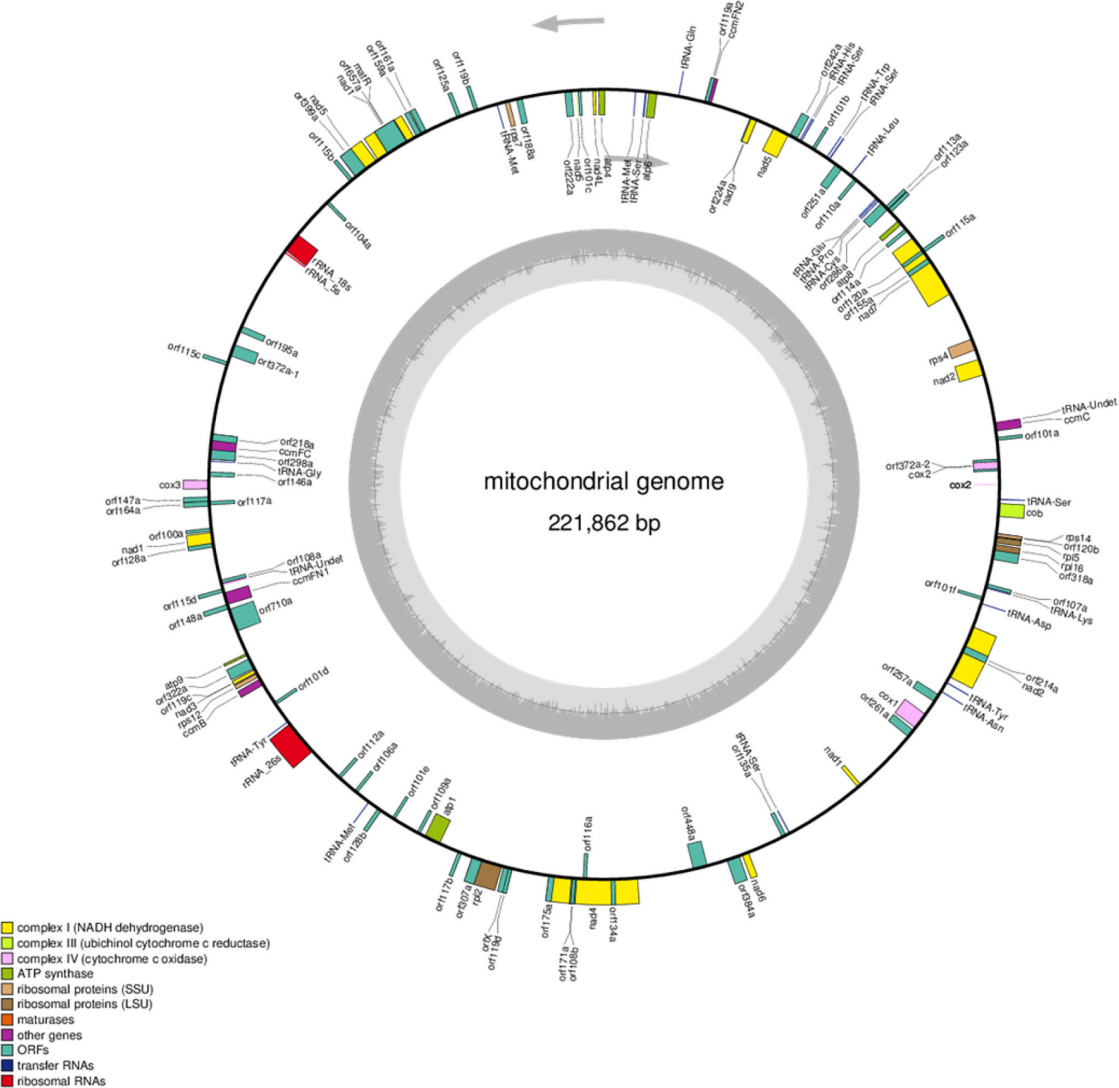
Mitochondrial genome map of the C5-type CMS line. Genes with names inside the circle are transcribed clockwise. Genes with names outside the circle are transcribed counterclockwise. The colors of the genes denote the functions of the gene products.

The mitochondrial genomes of the C5-type CMS cabbage line were sequenced with Illumina HiSeq and PacBio Sequel techniques. The Illumina HiSeq sequencing platform generated 6791 Mb of clean data. Then, 47.85 Mb of subread bases were produced for the sample in the PacBio sequencing platform. The mitochondrial genome of the C5-type CMS cabbage line was assembled into a single circular mapping molecule with a size of 221,862 bp (GenBank no. ON960289) (**Figure 2**). The G+C content of the C5-type CMS mitochondrial genome was 45.19%, which was comparable to that of our previously released mitochondrial genomes from maintainer and Ogura-type CMS cabbage lines. Additionally, the C5-type CMS mitochondrial genome possessed a total of 125 genes, including 99 protein-coding genes (32 known genes and 67 unknown-function ORFs), 3 rRNA genes (5S, 18S, and 26S), and 23 tRNA genes. A comparison between the maintainer and C5-type CMS mitochondrial genomes revealed that most known mitochondrial genes were identical. The C5-type CMS mitochondrial genome comprised three genes encoding the large subunits of ribosomal proteins (*rpl2*, *rpl5*, and *rpl16*), four genes encoding the small subunits of ribosomal proteins (*rps4*, *rps7*, *rps12*, and *rps14*), nine subunits of NADH dehydrogenase (*nad1*, *nad 2*, *nad* 3, *nad4*, *nad4L*, *nad*5, *nad*6, *nad*7, and *nad*9), three cytochrome oxidase subunits (*cox1*, *cox2*, and *cox3*), one cytochrome b (*cob*), five subunits of ATP synthase (*atp1*, *atp4*, *atp6*, *atp8*, and *atp9*), five cytochrome C synthesis-related genes (*ccmB*, *ccmC*, *ccmFN1*, *ccmFN2*, and *ccmFC*), and one maturase gene (*matR*).

### Collinearity analysis of the mitochondrial genomes

**Figure 3:**
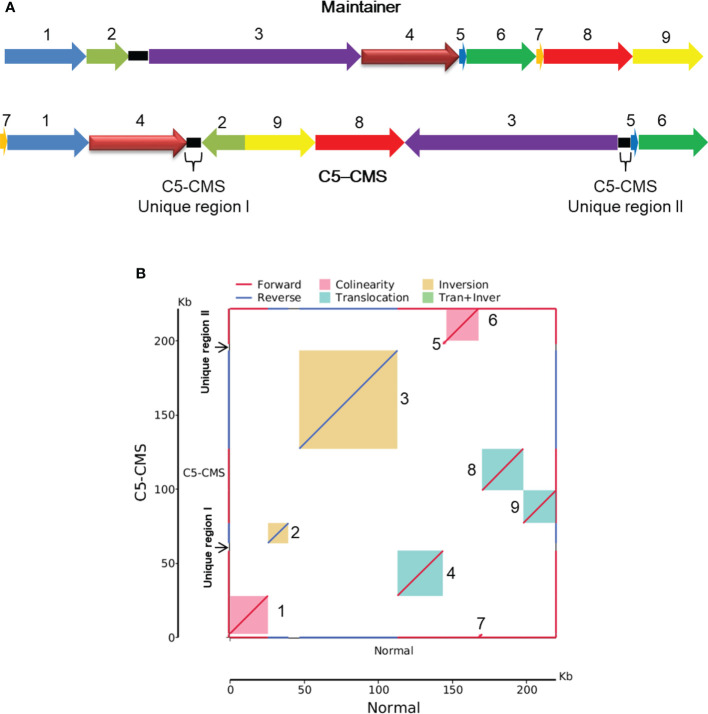
Collinearity analysis of the mitochondrial genomes between the maintainer and C5-type CMS lines. **(A)** Schematic illustration of 9 syntenic regions in the mitochondrial genomes of the maintainer and C5-CMS cabbage lines. The nine syntenic regions were named block 1 to block 9. The C5-CMS line had two unique regions. **(B)**. Maintainer line genome (X-axis) plotted against the C5-CMS line genome (Y-axis). Red lines indicate forward alignment, and blue lines indicate reverse alignment. The numbers behind the inner squares for the syntenic regions correspond to those indicated in panel **(A)** The color of the inner bars represents the alignment types. Red color: collinearity; green color: translocation; yellow color: inversion.

To further analyze the structure and composition of the C5-type CMS cabbage mitochondrial genome, collinearity analysis was performed with the maintainer line. Our sequencing results showed that the mitochondrial genome size of the C5-type CMS line (221,862 bp) was 1,900 bp larger than that of its maintainer line (219,962 bp). In addition, a total of 9 syntenic regions (named blocks 1-9) were identified in the mitochondrial genomes of the maintainer and C5-type CMS cabbage lines on the basis of sequence homology (**Figure 3A**). These regions ranged from 2,317 to 66,388 bp in length and accounted for 95.85% of the mitochondrial genome sequence (**Supplementary Table S2**). The corresponding blocks between C5-CMS and its maintainer line had at least 99.22% identity (**Supplementary Table S2**). Although all 9 syntenic regions shared high sequence similarity, the directions and positions of these syntenic regions differed between the two mitochondrial genomes. The orientation of seven regions was identical, but in the other two (block 2 and block 3), the blocks were inversions. Block 4, block 8, and block 9 were translocations (**Figure 3**). The syntenic regions were largely discrepant in the two genomic positions, although the genetic sequences were well conserved. Recombination and rearrangement events during the breeding process were determined to be responsible for the structural differences in the mitochondrial genomes of the same species. Block 4/block 2 and block 3/block 5 in the C5-CMS mitochondrial genome were broken by unique region I and unique region II, which were nonhomologous to the regions in the maintainer mitochondrial genome (**Figure 3**).

### Analysis of the unique regions in the C5-type CMS mitochondrial genome and phylogenetic analysis of mitochondrial genomes

**Figure 4:**
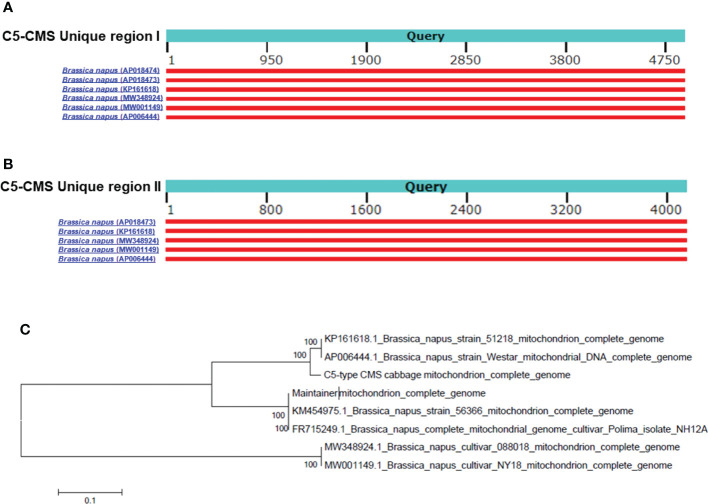
C5-CMS unique regions homologous to mitochondrial genomes and phylogenetic analysis based on the mitochondrial genome. Alignment of the C5-CMS unique region I **(A)** and C5-CMS unique region II **(B)** to the mitochondrial genomes of the *B. napus* lines (AP018474, AP018473, KP161618, MW348924, MW001149, and AP006444). The query sequences were C5-CMS unique region I (4897 bp) and unique region II (4141 bp). Red-colored boxes indicate the scores of sequence alignment with the mitochondrial genomes. **(C)** Phylogenetic tree constructed using C5-type CMS mitochondrial sequence and seven complete mitochondrial genome sequences of *B. napus* lines (AP018474, AP018473, KP161618, MW348924, MW001149, KM454975 and AP006444). The unrooted phylogenetic tree was created in MEGA 6.0 software by the ML method with 500 bootstrap iterations.

**Figure 5:**
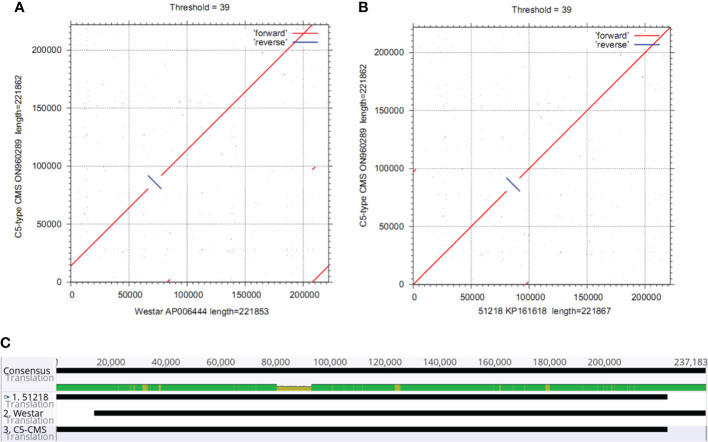
Comparative analysis of the mitochondrial genomes between the C5-type CMS line and the *B*. *napus* cultivars 51218 and Westar. **(A)** Dot plot alignment of the cabbage C5-type CMS and *B. napus* cultivar 51218 mitochondrial genomes. The comparisons show that the mitochondrial genome nucleotide sequences of *B. napus* cultivar 51218 (horizontal axis) resemble those of C5-type CMS cabbage (vertical axis). **(B)** Dot plot alignment of the C5-type CMS (vertical axis) and Westar (horizontal axis) mitochondrial genomes. The numbers indicate syntenic regions using the 51218 and Westar genome sequences as references. Comparisons showed that the mitochondrial genome sequence of the C5-type CMS line was similar to that of the *B*. *napus* cultivar 51218. **(C)** Pairwise alignment of mitochondrial genomes between the C5-type CMS line and *B. napus* cultivars 51218 and Westar was performed using Geneious Prime software. The sequence of the C5-type CMS mitochondrial genome was more homologous to that of 51218 than to that of Westar.

The collinearity analysis results revealed two unique regions in the C5-type CMS mitochondrial genome (**Figure 3A**). The sizes of unique region I and unique region II were 4,897 bp and 4,141 bp, respectively. The total length of the unique regions was 9,038 bp, accounting for 4.07% of the whole C5-type CMS mitochondrial genome. At the nucleotide level, BLASTn analysis showed that the unique regions shared high similarity with mitochondrial sequences of *B. napus* strain SW18 (AP018473 and AP018474), strain 51218 (KP161618), cultivar 088018 (MW348924), cultivar NY18 (MW001149), cultivar 56366 (KM454975), and cultivar Westar (AP006444) (**Figures 4A, B**). The results suggested that the C5-type CMS cytoplasm may have been derived from certain *B. napus* CMS haplotypes. Furthermore, phylogenetic analysis was performed using eight complete mitochondrial genomes. Whole-genome-wide alignments and maximum likelihood (ML) bootstrap analysis with 500 replicates were performed using MEGA 6.0. As shown in **Figure 4C**, the phylogenetic tree revealed the topological structure of eight selected taxa, which were mainly divided into three clades. The C5-type CMS mitochondrial sequence formed Clade I with *B. napus* strains 51218 and Westar, which are *nap*-type strains of *B. napus*. Clade II comprised the maintainer cabbage line and *B. napus* strains 56366 and NH12A, which are *pol*-type *B. napus*. Another two *nap*-type *B. napus* cultivars, 088018 and NY18, constituted Clade III. Four *nap*-type accessions were clustered into two different clades, indicating that the investigated *nap*-type *B. napus* strains have a polyphyletic maternal origin. To further compare the mitochondrial genomes between C5-type CMS and *B. napus* cultivars 51218 and Westar, dot plot analysis and pairwise alignment were performed with Geneious Prime software. The results showed that the sequence of the C5-type CMS mitochondrial genome was more homologous to that of the *B. napus* cultivar 51218 than to that of Westar. However, there was an inversion located at the position from 80,500 bp to 93,000 bp, and many single-nucleotide polymorphisms (SNPs) were distributed throughout the genome (**Figure 5**). Taken together, these results show that the C5-type CMS original cytoplasm donor material was most likely generated by intergeneric hybridizations with certain *nap*-type CMS *B. napus* strains during the breeding process.

### Identification of CMS-associated ORFs in the C5-type CMS mitochondrial genome

**Figure 6:**
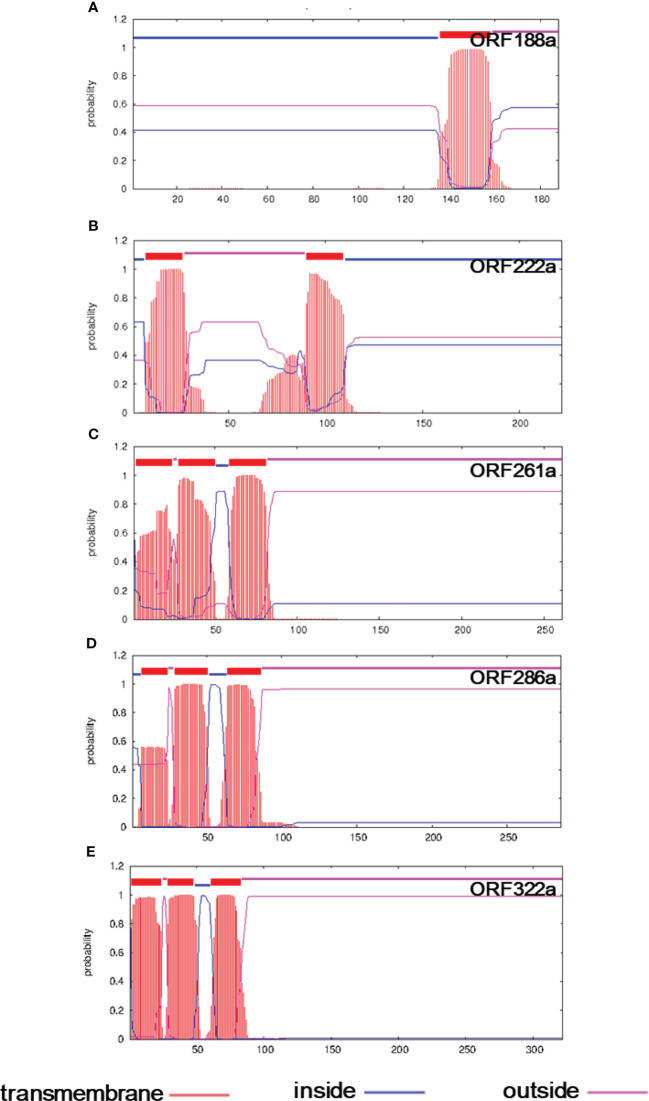
Transmembrane domain prediction of candidate CMS gene-encoded proteins in the C5-CMS line. The output of the TMHMM server indicated the location and probability associated with the predicted transmembrane domains in the C5-CMS line. **(A)** ORF188a, **(B)** ORF222a, **(C)** ORF261a, **(D)** ORF286a, and **(E)** ORF322a.

To reveal the genes determining the CMS phenomenon, we compared the mitochondrial genomes between the C5-type CMS line and its maintainer lines. Genome rearrangement events may have caused the presence of variation in genome sequence segments. Specific genes were generated by recombination, which is generally responsible for the CMS trait. Seven ORFs that encoded over 100 amino acids, including *orf114a*, *orf123a*, *orf188a*, *orf222a*, *orf261a*, *orf286a*, and *orf322a*, were specifically identified in the C5-type CMS mitochondrial genome (**Table 1**). All of these specific ORFs were candidate CMS genes. To further verify whether the proteins encoded by these ORFs were candidate proteins for CMS, the structures of these three proteins were predicted. We found that ORF188a, ORF222a, ORF261a, ORF286a, and ORF322a had transmembrane domains, whereas ORF114a and ORF 123a lacked transmembrane domains (**Figure 6**).

**Table 1:** Specific ORFs in the Ogura-CMS mitochondrial genome.

Specific ORFs	Most similar mitochondrial sequence of another species	Location in the C5-type CMS line
orf114a	YP_717103.1 hypothetical protein BrnapMp004 [*Brassica napus*]	Block 1
orf123a	AKD00165.1 hypothetical protein [*Brassica napus*]	Block 1
orf188a	YP_717121.1 hypothetical protein BrnapMp023 [*Brassica napus*]	Unique region I
orf222a	YP_717120.1 hypothetical protein BrnapMp022 [*Brassica napus*]	Between block 4 and unique region I
orf261a	YP_717164.1 hypothetical protein BrnapMp067 [*Brassica napus*]	Between unique region II and block 5
orf286a	YP_717106.1 hypothetical protein BrnapMp007 [Brassica napus]	Between block 1 and block 4
orf322a	YP_717145.1 hypothetical protein BrnapMp048 [*Brassica napus*]	Between block 8 and block 3

Seven ORFs, including ORF114a, ORF123a, ORF188a, ORF222a, ORF261a, ORF286a, and ORF322a, were specifically identified in the C5-type CMS mitochondrial genome. They are identical to the mitochondrial proteins of Brassica napus. ORF144a and ORF123a were located in C5-type CMS block 1, ORF188a was located in C5-type CMS unique region I, ORF222a was located between C5-type CMS block 4 and unique region I, ORF261a was located between C5-type CMS unique region II and block 5, ORF286a was located between block 1 and block 4, and ORF322a was located between block 8 and block 3.

### Organization of mitochondrial genome regions associated with candidate sterility genes for C5-type CMS

**Figure 7:**
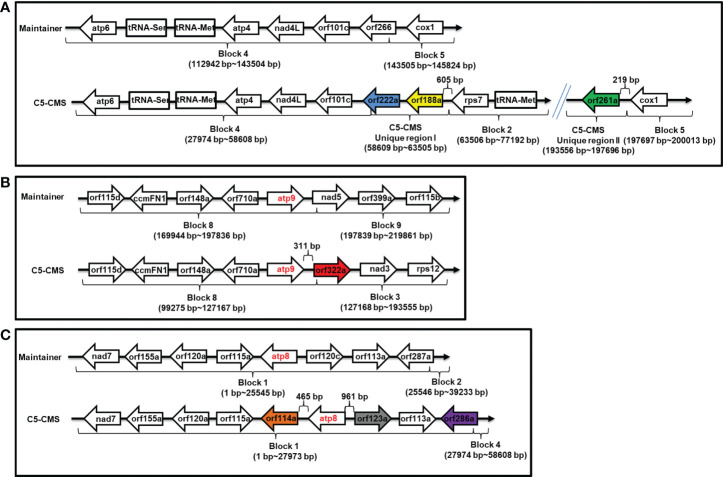
Organization of mitochondrial genome regions associated with candidate sterility genes for C5-type CMS. **(A)** Genome structure of the orf188a, orf222a, and orf261a regions. **(B)** Genome structure of the atp9 and orf322a regions. **(C)** Genome structure of the atp8, orf114a, orf123a and orf286a regions. The corresponding genes within the regions between the maintainer and C5-type CMS genomes areindicated by boxes. Two recombination events occurred in the C5-type CMS genome. Unique region I was inserted between the block 4 and block 2 syntenic regions, and orf188a and orf222a are located in unique region I. Unique region II was the result of recombination between the block 3 and block 5 syntenic regions, and orf261a is located in this region.

A more detailed analysis of the organization of mitochondrial genome regions associated with candidate sterility genes for C5-CMS was carried out. *orf188a* was located in C5-type CMS unique region I 651 bp downstream of *rps7*, and *orf222a* was located between C5-CMS block 4 and unique region I (**Supplementary Table S3** and **Figure 7A**); however, *orf261a* was located 219 bp downstream of *cox1* between C5-CMS unique region II and block 5 (**Supplementary Table S3** and **Figure 7A**). *orf322a* was located 311 bp downstream of *atp9* between block 8 and block 3, and *orf114a* and *orf123a* were located 465 bp downstream and 961 bp upstream of *atp8* within block 1, respectively (**Supplementary Table S3**, **Figures 7B, C**). However, *orf286a* was located 1,324 bp upstream of *atp8* between block 1 and block 4 of the C5-type CMS mitochondrial genome. Taken together, the findings indicate that the CMS candidate genes *orf114a*, *orf188a*, *orf261a*, and *orf322a* are cotranscribed with *atp8*, *rps7*, *cox1*, and *atp9*, respectively.

### ORF222a affects F_1_F_0_-ATP synthase assembly by interacting with an ATP17 homolog (ATP17-2, *Bo7g114140*) in *B. oleracea*

**Figure 8:**
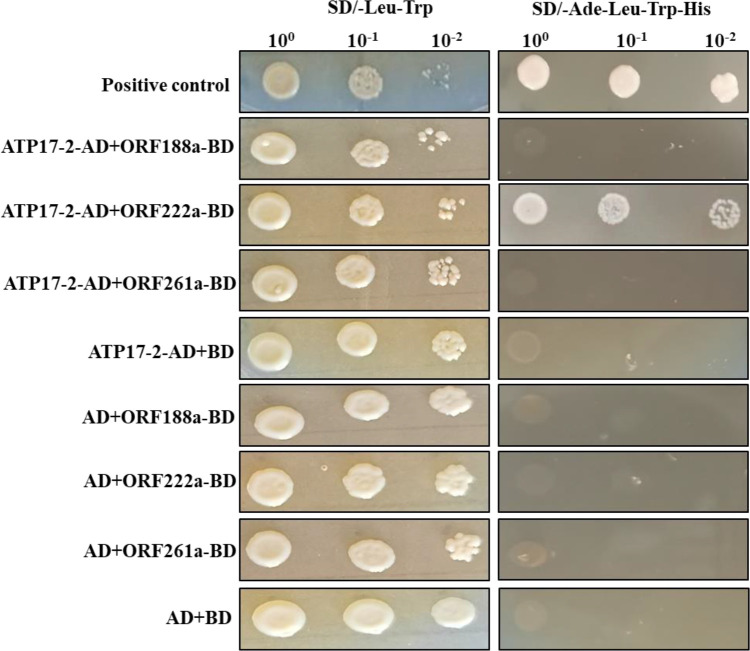
Y2H assay of the interaction of ORF222a with ATP17-2. Serial dilutions of cotransformed yeast were spotted on synthetic dropout SD/-Trp-Leu medium to select for cotransformants and SD/-Trp-Leu-His-Ade medium to select for positive interactions. p53 was used as a positive control.

To determine whether candidate CMS-associated ORF-encoded proteins (ORF188a, ORF222a and ORF261a) were capable of binding to subunits of F_1_F_0_-ATP synthase, a yeast two-hybrid (Y2H) assay was performed with ORF-encoded proteins (ORF188a, ORF222a, and ORF261a) and subunits (ATP4, ATP6, ATP8, ATP9, and ATP17) of F-ATP synthase. *orf188a*, *orf222a*, and *orf261a* were cloned into the PGBKT7 vector; meanwhile, *atp4*, *atp6*, *atp8*, *atp9*, and two *atp17*-homologous genes (*atp17-1*, *Bo3g175820* and *atp17-2*, *Bo7g114140*) were cloned into PGADT7 vectors. Eighteen candidate interaction combinations were tested with the Y2H assays. The results showed that only ORF222a interacted with the ATP17 homolog (ATP17-2, *Bo7g114140*) in *B. oleracea* (**Figure 8** and **Supplementary Figure S2**). We thus conclude that ORF222a impairs the step of F_1_Fo-ATP synthase assembly by interacting strongly with the ATP17 homolog (ATP17-2, *Bo7g114140*) in *B. oleracea*.

### Detection of ATP production in the C5-type CMS cabbage line

**Figure 9:**
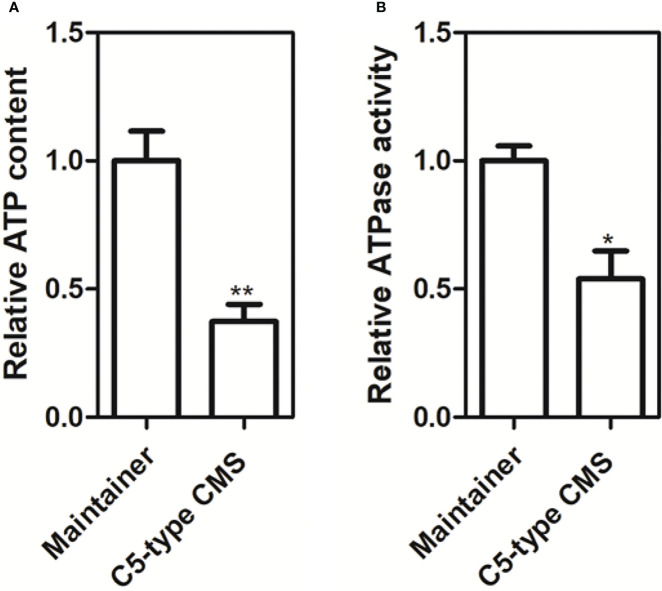
Production of ATP and ATP synthase activity in anther samples of maintainer and Ogura-CMS cabbage lines. **(A)** Comparison of the ATP content in petals of the maintainer and C5-type CMS cabbage lines. **(B)** Comparison of ATP synthase activity in petals of the maintainer and C5-type CMS cabbage lines. The *t* test comparison statistics are shown (means ± SDs; n = 3). *P < 0.05; **P < 0.01.

From our whole-mitochondrial genome data, we found that the C5-type CMS-related gene *orf188a* encodes the *atp6*-like gene and that *orf222a* encodes 58 amino acids identical to ATP8 at the N-terminus (**Supplementary Figure S1**). We also found that *orf188a*, *orf261a*, and *orf322a* are cotranscribed with *rps7*, *cox1*, and *atp9*, respectively. Furthermore, ORF222a interacts with an ATP17 homolog (*Bo7g114140*) in *B. oleracea*. Therefore, we wondered whether these events influence the yield of ATP. We measured the ATP content and ATPase activity in anther samples from maintainer and C5-type CMS cabbage lines. We found that the ATP content and ATPase activity in the anther samples of the C5-type CMS cabbage line was remarkably lower than that in the maintainer line (**Figure 9**). The change in ATP yield may have been caused by altered ATPase activity. In conclusion, CMS-related genes encode partial fragments identical to mitochondrial genes with known functions and are cotranscribed with or interact with these mitochondrial electron transport chain (mtETC) genes, which might disturb mitochondrial energy metabolic pathways.

### Cytological analysis of anther development between the maintainer and C5-type CMS cabbage lines

**Figure 10:**
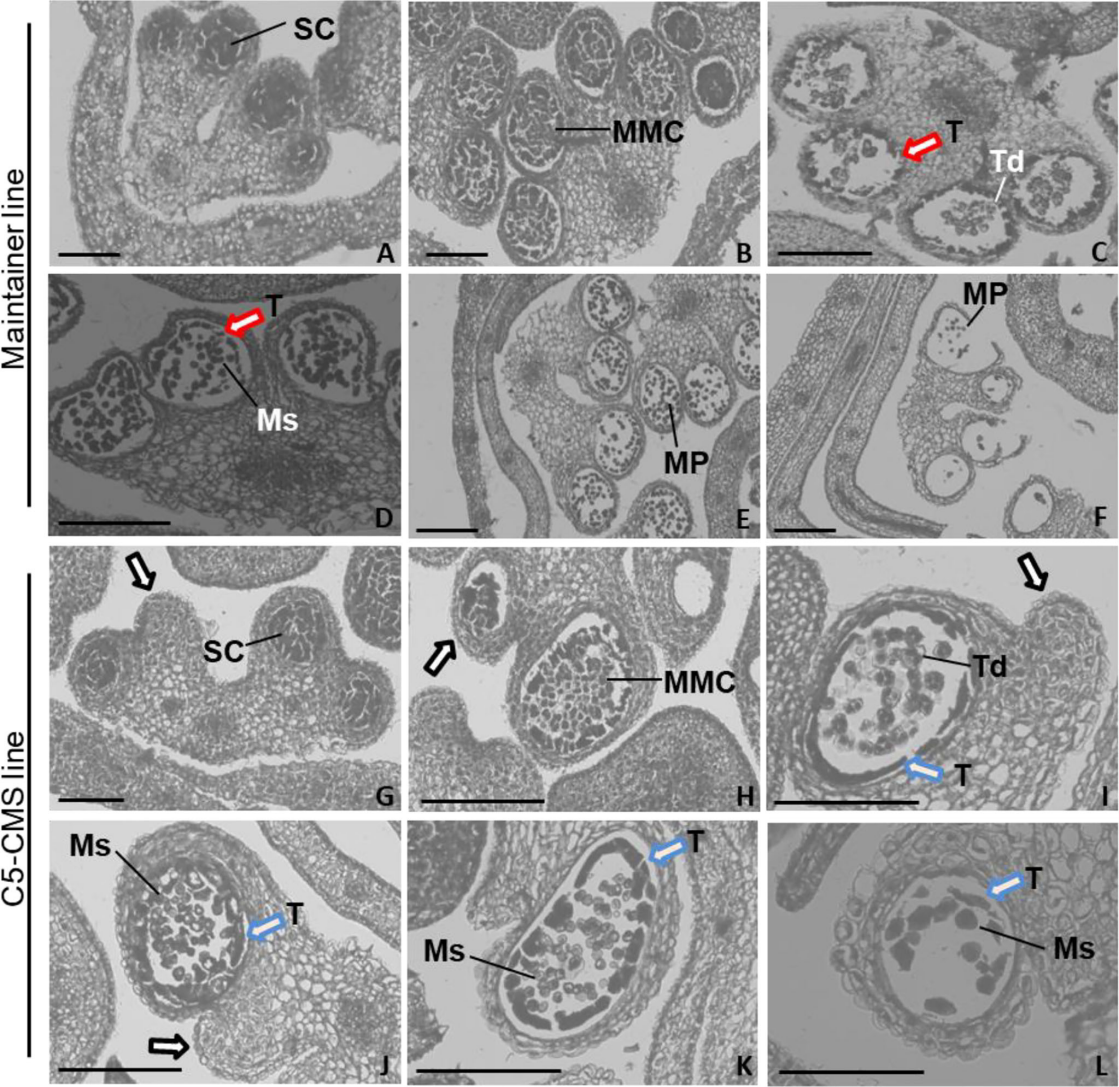
Comparative histology of anther development in *B*. *oleracea* maintainer and C5-type CMS lines. Histology of anther development in the maintainer line **(A–F)** and C5-type CMS line **(G–L)**. **(A, G)** are the sporogenesis cell stage; **(B, H)** are the microspore mother cell stage; **(C, I)** are the tetrad stage; **(D, J)** are the uninucleate microspore stage; **(E, K)** are the mature pollen stage; and **(F, L)** are the anthesis stage. SC, sporogenesis cell; MMC, microspore mother cells; T, tapetum; Td, tetrad; Ms, microspore; MP, mature pollen. Bar = 200 um.

Our mitochondrial sequence data indicated that the C5-type CMS cabbage line possesses CMS genes from the *nap-*type CMS system of *B. napus*. However, the mechanism by which male sterility genes induce pollen abortion in cabbage is still uncertain. Thus, comparative cytological analysis of anther development between the maintainer and C5-type CMS cabbage lines was performed to address which developmental stage was affected in the stamens of the C5-type CMS line and to further determine the cause of C5-type CMS in cabbage. In the maintainer line, primordial anther tissue with four microsporangiums was found to differentiate into sporogenous cells (**Figure 10A**), which differentiated into irregularly shaped microspore mother cells. The tapetal cells, middle layer, endothecium and epidermis differentiated from the surrounding connective tissue (**Figure 10B**). Tetrads of microspores were generated after meiotic divisions, the tapetal cells began to degrade (marked by the red arrow) (**Figure 10C**). Free haploid microspores dissociated from the tetrads during the uninucleate microspore stage, and the most of tapetal cells degrade (**Figure 10D**). The microspores differentiated into mature pollen grains, and the tapetal cells degenerated completely (**Figure 10E**). Finally, the mature pollen grains were released from the dehiscent anther (**Figure 10F**). In the maintainer cabbage line, four locules of anthers developed symmetrically (**Figures 10A–F**). In contrast, a loss of synchronous locule development was observed in the C5-type CMS anthers during all anther development stages (marked by the black arrows) (**Figures 10G–L**). There were no morphological differences in the developed locules between the maintainer and C5-type CMS cabbage lines at the sporogenesis cell stage and microspore mother cell stage (**Figures 10A, B**, **10G, H**). However, the tapetum prematurely separated from the locule wall at the tetrad stage in the C5-type CMS line (marked by the blue arrows) (**Figure 10I**), rather than separating from the connective tissue during the uninucleate microspore stage, as in the maintainer cabbage line (**Figure 10D**). In the uninucleate microspore stage of the C5-type CMS line, haploid microspores formed but crowded together (**Figure 10J**). The tapetal cells were still quite intact at the uninucleate microspore stage and mature pollen stage in the C5-type CMS line (marked by the blue arrows) (**Figures 10J, K**). Premature separation of the tapetum from the connective tissue and delayed cell collapse of tapetal cells hindered the release of haploid microspores, which resulted in the structural collapse of microspores. The residues of microspores that failed to develop were visible, and the epidermis, fibrous layer, and intermediate layer of pollen sacs were tightly connected, generally without dehiscence (**Figures 10K, L**). The tapetal cellular debris was still observed at the anthesis stage (marked by the blue arrow) (**Figure 10L**). Taken together, these findings indicate that premature separation of the tapetum from the locule wall and abnormal degradation of tapetal cells lead to overlap of microspores, which might be the cause of CMS in C5-type CMS cabbage lines.

## Discussion

### Mitochondrial origin of *nap*-type CMS in C5-type CMS *B. oleracea*

Most CMS systems of *B. oleracea* (cabbage) were transferred from other cruciferous crops. Because of incomplete pollen abortion and abnormal flower organ growth, the *pol* and *Nig* CMS systems cannot be used for cabbage breeding. The Ogu-CMS system is the main type of male sterility system in cabbage breeding (Ji et al., 2020). However, a high degree of cytoplasmic genetic uniformity may lead to genetically vulnerable plants (Yamagishi and Terachi, 2017), which prompted us to generate and apply a new type of CMS in cabbage. In this study, we generated the C5-type CMS cabbage line *via* intergeneric hybridization with *nap*-type CMS *B. napus* and then consecutive backcrossing. Sequencing revealed that the mitochondrial genome of the C5-type CMS cabbage line is quite homologous to that of the *nap*-type CMS *B. napus* strains 51218 and Westar but significantly different from that of common cabbage (**Figures 3B**, **4C**, **5**). Both the chloroplast and mitochondrial genomes of progeny by sexual propagation are usually identical to those of maternal parents without recombination (Park et al., 2021). Here, the original cytoplasm of C5-type CMS cabbage was inherited from a certain *nap*-like CMS *B. napus* haplotype. However, the mitochondrial genome sequence of this *B. napus* haplotype has not yet been released.

### C5-type CMS is associated with energy deficiency

Many ORFs in CMS lines have been found to disrupt energy metabolism through cotranscription with the F_1_F_0_-ATP synthase subunit. Here, we found that *orf188a*, *orf322a*, and *orf322a* were cotranscribed with *rps7*, *cox1*, and *atp9*, respectively (**Figure 7**). Most CMS-related genes have been found to be chimeric ORFs consisting of fragments of coding sequences of known mitochondrial genes from mtETC pathways, including *cox1*, *atp6*, *atp8*, *atp9*, and some other mitochondrial genes (Chen and Liu, 2014). For instance, *orf463a* for radish DCGMS-type CMS possesses a 128 bp fragment corresponding to part of the *cox1* sequence (Wang et al., 2020). *orf346* for *Nsa*-type CMS also shows partial sequence identity with the *cox1* gene (Sang et al., 2021). In our previous study, we found that the Ogura*-*type CMS *B. oleracea* gene *orf138*, which originated from radish, encodes an *atp8*-like gene. The CMS-related gene *orf154a* is partially homologous to the *ATP synthase subunit 1* (*atpA*) gene (Zhong et al., 2021). In *nap*-type CMS *Brassica*, chimeric *orf222* encodes a protein with a segment of ATP8 at the N-terminus plus a sequence of unknown origin (Geddy et al., 2005). *orf288* for *Hau-*type CMS *B. juncea* contains a 94 bp sequence with partial sequence homology to *nad5* and partial sequence identity to *atp9* (Heng et al., 2014). In our results, we found that the C5-type CMS-related gene *orf188a* encodes the *atp6*-like gene and that *orf222a* encodes 58 amino acids identical to ATP8 at the N-terminus (**Supplementary Figure S1**). The CMS-related genes encode partial fragments consistent with known functional mitochondrial genes. It has been speculated that these chimeric genes and the cotranscription events may disrupt mitochondrial functions by interfering with the expression of native mitochondrial genes, affecting the assembly or activity of different mitochondrial complexes (Shaya et al., 2012). In addition, it has been reported that chimeric genes confer the CMS phenotype by directly interacting with genes involved in the mtETC or ATP synthase complex. In maize, the chimeric gene *atp6c* confers CMS by interacting with ATP8 and ATP9, which impairs the assembly of the mitochondrial ATP synthase complex. Ultimately, this results in reductions in the quantity and activity of assembled ATP synthase required for anther development (Yang et al., 2022). In our study, we found that ORF222a impaired the step of F_1_F_0_-ATP synthase assembly by interacting strongly with an ATP17 homolog (*Bo7g114140*) in *B. oleracea* (**Figure 8** and **Supplementary Figure S2**). We also found that the ATP and ATPase activity levels in the anther samples of the C5-type CMS cabbage line were remarkably lower than those in the maintainer line (**Figure 9**). In addition, cytological sections showed that four locules develop synchronously through all stages of maintainer anther development (**Figures 10A–F**). In contrast, C5-type CMS anthers can display a loss of synchronous locule development (**Figures 10G–J**), which could be triggered by energy deficiency. Taken together, all of these findings indicate that C5-type CMS is associated with energy deficiency.

### The abnormal development of tapetal cells probably causes male sterility in the C5-type CMS cabbage line

Programmed cell death (PCD) of the tapetum is one of the most critical steps for fertility. It is generally assumed that abnormal tapetal cells fail to produce enough nutrients and enzymes for microspore development and release from the tetrad, which often triggers the abortion of microspores (Liu et al., 2015). Additionally, defective tapetum PCD has previously been reported to cause anther indehiscence in an autophagy-deficient mutant of rice (Kurusu et al., 2014). In our results, the premature separation of the tapetum from the connective tissue and delayed cell collapse of tapetal cells hindered the release of haploid microspores, which directly resulted in both the structural collapse of microspores and anther indehiscence in the C5-type CMS line (**Figure 10**). This result is consistent with the development of *nap*-type CMS anthers in the *Brassica napus* (Geddy et al., 2005). However, it is quite different from the case in the Ogura-type CMS cabbage line, in which abnormal proliferation of tapetal cells hinders and destroys the development of haploid microspores through spatial constriction (Zhong et al., 2021).

## Conclusion

We generated a new type of CMS cabbage line, named the C5-type CMS line, which showed abortion of microspores and anther indehiscence. *orf188a*, *orf222a*, *orf261a*, *orf286a*, and *orf 322a* were specifically identified as candidate CMS genes *via* sequencing of the mitochondrial genome of the C5-type CMS cabbage line. We found that *orf114a*, *orf188a*, *orf261a* and *orf322a* were cotranscribed with *atp8*, *rps7*, *cox1*, and *atp9*, respectively (**Figure 7**). The most interesting finding was that ORF222a interacts with an ATP17 homolog (*Bo7g114140*) in *B. oleracea*, which might impair the step of F_1_Fo-ATP synthase assembly. We also found that the ATP and ATPase activity levels in the anther samples of the C5-type CMS cabbage line were remarkably lower than those in the maintainer line. These candidate CMS genes may perturb ATP synthesis during the anther development, which triggers a loss of synchronous locule development, premature separation of the tapetum from the connective tissue, and delayed cell collapse of tapetal cell in anthers, finally leading to CMS.

## Data availability statement

The data presented in the study are deposited in the NCBI repository, accession number ON960289. The data will be released on Sep 17, 2022. We also provide confirmation of deposition.

## Author contributions

XZ carried out the sequence data analysis, and drafted the manuscript. XZ, XY, JC, RH and YG designed and coordinated all experiments. JK supervised the work and edited the manuscript. All authors read and approved the final manuscript.

## Funding

This study was supported by the National Natural Science Foundation of China (31972408) and the Science and Technology Innovation Capacity Building Projects of the Beijing Academy of Agriculture and Forestry Sciences (KJCX20200410, KJCX20210425, and KJCX20200113).

## Acknowledgments

We thank Dr. Qingbiao Wang from Beijing Vegetable Research Center, Beijing Academy of Agriculture and Forestry Sciences, for his helpful suggestion for our manuscript.

## Conflict of interest

The authors declare that the research was conducted in the absence of any commercial or financial relationships that could be construed as a potential conflict of interest.

## Publisher’s note

All claims expressed in this article are solely those of the authors and do not necessarily represent those of their affiliated organizations, or those of the publisher, the editors and the reviewers. Any product that may be evaluated in this article, or claim that may be made by its manufacturer, is not guaranteed or endorsed by the publisher.
