# Supplementary material for: Complete mitochondrial genome sequencing and identification of candidate genes responsible for C5-type cytoplasmic male sterility in cabbage (B. oleracea var. capitata)
Source: Front Plant Sci. 2022 Sep 26;13:1019513. doi: 10.3389/fpls.2022.1019513 (PMC9549296; doi:10.3389/fpls.2022.1019513)
Supplement: Supplementary file 1 [file Presentation_1.pptx]

## Slide 1
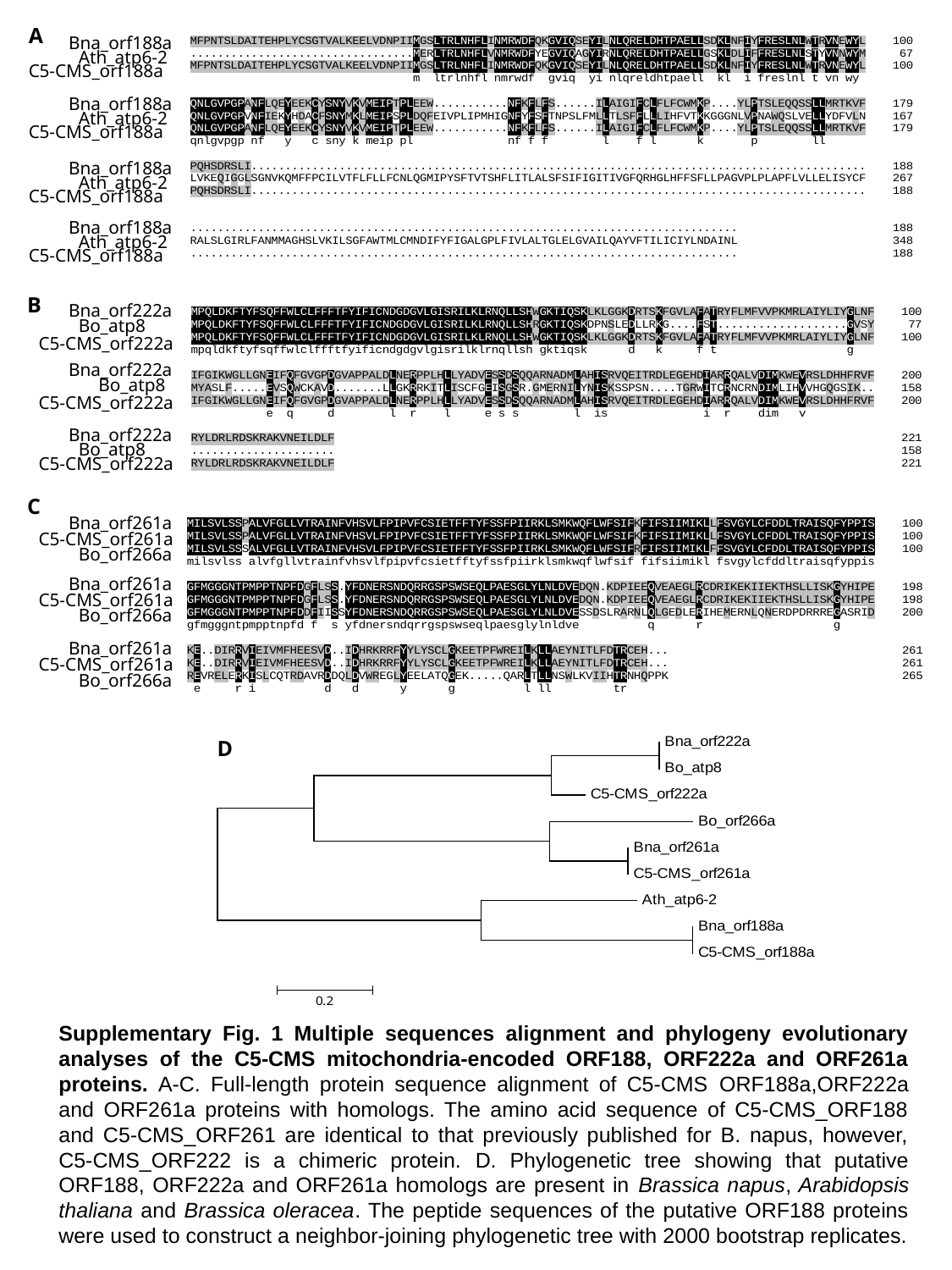

A
Bna_orf188a
Ath_atp6-2
C5-CMS_orf188a
Bna_orf188a
Ath_atp6-2
C5-CMS_orf188a
Bna_orf188a
Ath_atp6-2
C5-CMS_orf188a
Bna_orf188a
Ath_atp6-2
C5-CMS_orf188a
B
Bna_orf222a
Bo_atp8
C5-CMS_orf222a
Bna_orf222a
Bo_atp8
C5-CMS_orf222a
Bna_orf222a
Bo_atp8
C5-CMS_orf222a
C
Bna_orf261a
C5-CMS_orf261a
Bo_orf266a
Bna_orf261a
C5-CMS_orf261a
Bo_orf266a
Bna_orf261a
C5-CMS_orf261a
Bo_orf266a
D
Supplementary Fig. 1 Multiple sequences alignment and phylogeny evolutionary analyses of the C5-CMS mitochondria-encoded ORF188, ORF222a and ORF261a proteins. A-C. Full-length protein sequence alignment of C5-CMS ORF188a,ORF222a and ORF261a proteins with homologs. The amino acid sequence of C5-CMS_ORF188 and C5-CMS_ORF261 are identical to that previously published for B. napus, however, C5-CMS_ORF222 is a chimeric protein. D. Phylogenetic tree showing that putative ORF188, ORF222a and ORF261a homologs are present in Brassica napus, Arabidopsis thaliana and Brassica oleracea. The peptide sequences of the putative ORF188 proteins were used to construct a neighbor-joining phylogenetic tree with 2000 bootstrap replicates.
