# Supplementary material for: Complete mitochondrial genome sequencing and identification of candidate genes responsible for C5-type cytoplasmic male sterility in cabbage (B. oleracea var. capitata)
Source: Front Plant Sci. 2022 Sep 26;13:1019513. doi: 10.3389/fpls.2022.1019513 (PMC9549296; doi:10.3389/fpls.2022.1019513)
Supplement: Supplementary file 2 [file Presentation_2.pptx]

## Slide 1
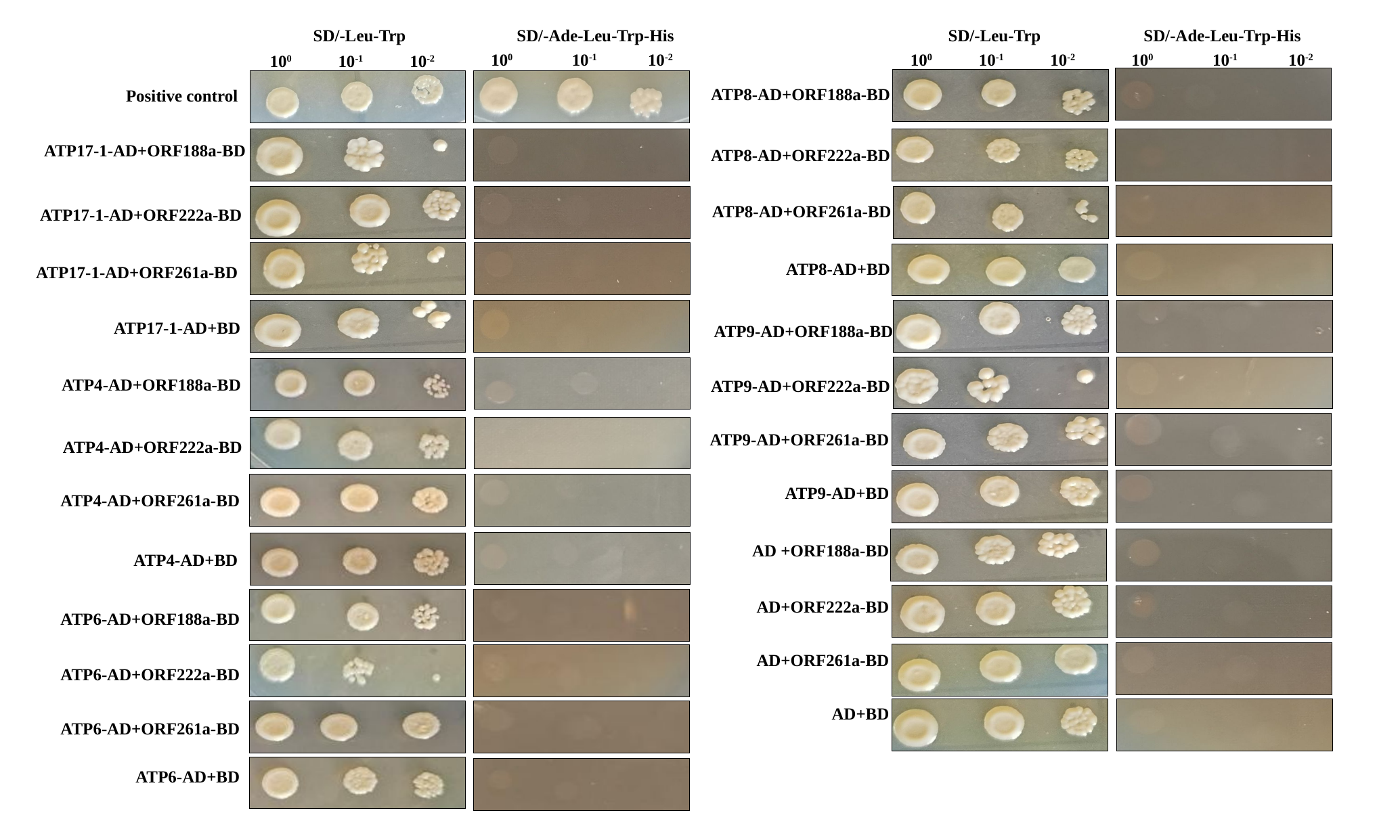

SD/-Leu-Trp
SD/-Ade-Leu-Trp-His
Positive control
ATP17-1-AD+ORF188a-BD
ATP17-1-AD+ORF222a-BD
ATP17-1-AD+ORF261a-BD
ATP17-1-AD+BD
ATP4-AD+ORF188a-BD
ATP4-AD+ORF222a-BD
ATP4-AD+ORF261a-BD
ATP4-AD+BD
ATP6-AD+ORF188a-BD
ATP6-AD+ORF222a-BD
ATP6-AD+ORF261a-BD
ATP6-AD+BD
100 10-1 10-2
100 10-1 10-2
SD/-Leu-Trp
SD/-Ade-Leu-Trp-His
ATP8-AD+ORF188a-BD
ATP8-AD+ORF222a-BD
ATP8-AD+ORF261a-BD
ATP8-AD+BD
ATP9-AD+ORF188a-BD
ATP9-AD+ORF222a-BD
ATP9-AD+ORF261a-BD
ATP9-AD+BD
AD +ORF188a-BD
AD+ORF222a-BD
AD+ORF261a-BD
AD+BD
100 10-1 10-2
100 10-1 10-2

## Slide 2
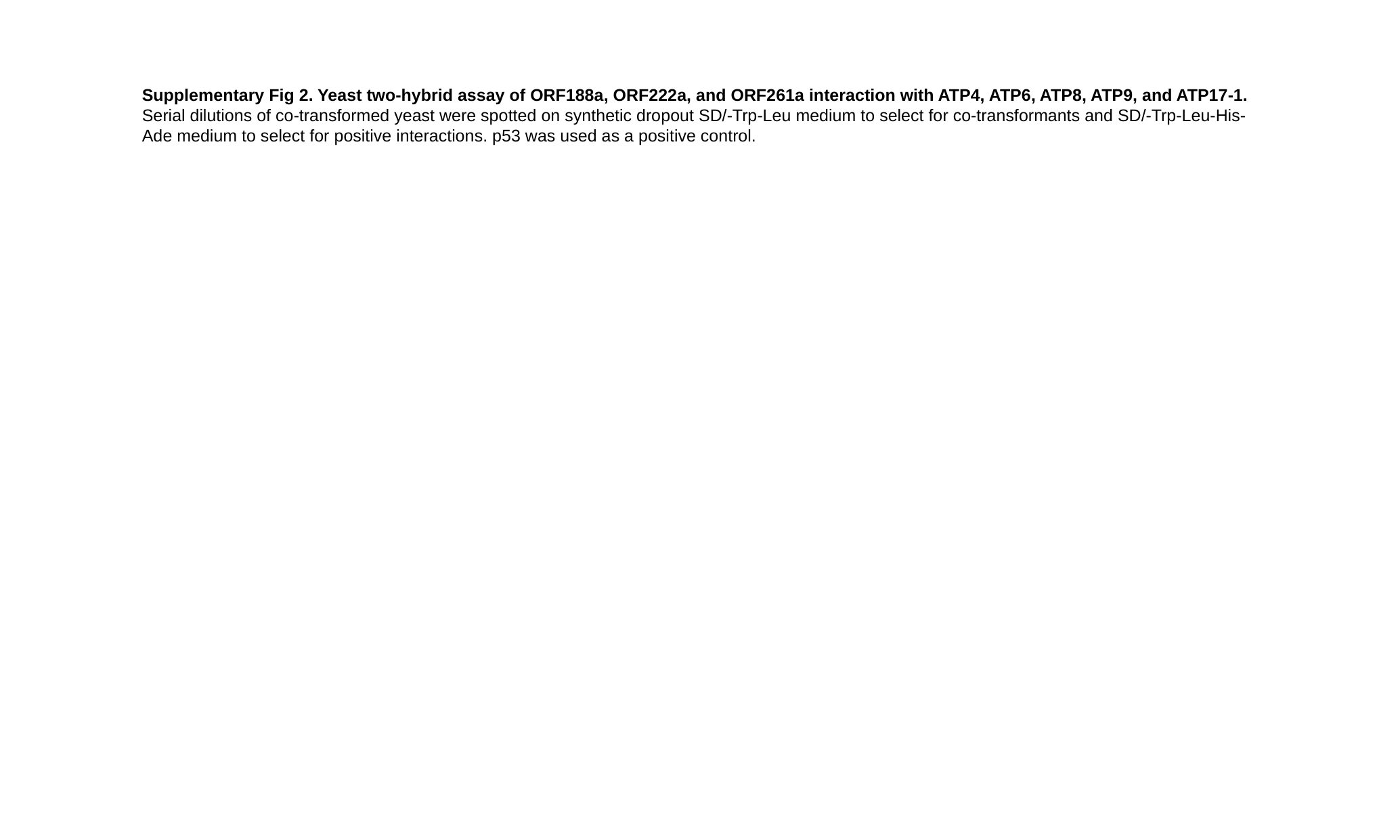

Supplementary Fig 2. Yeast two-hybrid assay of ORF188a, ORF222a, and ORF261a interaction with ATP4, ATP6, ATP8, ATP9, and ATP17-1. Serial dilutions of co-transformed yeast were spotted on synthetic dropout SD/-Trp-Leu medium to select for co-transformants and SD/-Trp-Leu-His-Ade medium to select for positive interactions. p53 was used as a positive control.
